# Ubc9 Attenuates Myocardial Ischemic Injury Through Accelerating Autophagic Flux

**DOI:** 10.3389/fphar.2020.561306

**Published:** 2020-09-15

**Authors:** Qing Xiao, Xiu-Hui Chen, Ru-Chao Jiang, Sheng-Ying Chen, Kai-Feng Chen, Xiang Zhu, Xiao-ling Zhang, Jun-jun Huang, Yuan Qin, Gui-Ping Zhang, Quan Yi, Jian-dong Luo

**Affiliations:** ^1^ The Fifth Affiliated Hospital, Key Laboratory of Molecular Target & Clinical Pharmacology and the State Key Laboratory of Respiratory Disease, School of Pharmaceutical Sciences, Guangzhou Medical University, Guangzhou, China; ^2^ Department of Pharmacology, Guangzhou Medical University, Guangzhou, China; ^3^ Guangzhou Institute of Cardiovascular Disease, Guangzhou Key Laboratory of Cardiovascular Disease, The Second Affiliated Hospital, Guangzhou Medical University, Guangzhou, China; ^4^ Department of Neonatology, Maternal and Children Hospital of Guangdong Province, Guangzhou, China

**Keywords:** Ubc9, SUMOylation, myocardial ischemia, autophagy, PI3K-Ⅲ complexes

## Abstract

**Aims:**

SUMOylation is a post-translational modification that plays a crucial role in the cellular stress response. We aimed to demonstrate whether and how the SUMO E2 conjugation enzyme Ubc9 affects acute myocardial ischemic (MI) injury.

**Methods and Results:**

Adenovirus expressing Ubc9 was administrated by multipoint injection in the border zone of heart immediately after MI in C57BL/6 mice. Neonatal rat cardiomyocytes (NRCMs) were also infected, followed by oxygen and glucose deprivation (OGD). *In vivo*, Ubc9 adenovirus-injected mice showed decreased cardiomyocyte apoptosis, reduced myocardial fibrosis, and improved cardiac function post-MI. *In vitro*, overexpression of Ubc9 decreased cardiomyocyte apoptosis, whereas silence of Ubc9 showed the opposite results during OGD. We next found that Ubc9 significantly decreased the accumulation of autophagy marker p62/SQSTM, while the LC3 II level hardly changed. When in the presence of bafilomycin A1 (BAF), the Ubc9 adenovirus plus OGD group presented a higher level of LC3 II and GFP-LC3 puncta than the OGD group. Moreover, the Ubc9 adenovirus group displayed increased numbers of yellow plus red puncta and a rising ratio of red to yellow puncta on the mRFP-GFP-LC3 fluorescence assay, indicating that Ubc9 induces an acceleration of autophagic flux from activation to degradation. Mechanistically, Ubc9 upregulated SUMOylation of the core proteins Vps34 and Beclin1 in the class III phosphatidylinositol 3-kinase (PI3K-III) complexes and boosted the protein assembly of PI3K-III complex I and II under OGD. Moreover, the colocalization of Vps34 with autophagosome marker LC3 or lysosome marker Lamp1 was augmented after Ubc9 overexpression, indicating a positive effect of Ubc9-boosted protein assembly of the PI3K-III complexes on autophagic flux enhancement.

**Conclusions:**

We uncovered a novel role of Ubc9 in protecting cardiomyocytes from ischemic stress *via* Ubc9-induced SUMOylation, leading to increased PI3K-III complex assembly and autophagy-positioning. These findings may indicate a potential therapeutic target, Ubc9, for treatment of myocardial ischemia.

## Introduction

Although there has been significant progress made in invasive heart-treatment strategies (i.e., percutaneous coronary intervention and intensive pharmacotherapy treatment), myocardial infarction (MI) and subsequent heart failure remain associated with a high risk of mortality (De et al., 2017). The process of post-infarction cardiac remodeling is rather complicated (Shetelig et al., 2018). Cardiomyocyte necrosis, excessive cardiomyocyte apoptosis, various cytokines, interstitial fibrosis, and dynamics of non-cardiac cells within ischemic tissues are all closely related to cardiac remodeling following MI (Liehn et al., 2011; Hind et al., 2012; Henri et al., 2016).

During myocardial ischemia and subsequent heart failure, protein aggregates and damaged or excess organelles are accumulated, which provokes an adaptive autophagic response to resist acute ischemic cell death. However, most reports demonstrate that basal autophagy is insufficient for aggregate and organelle clearance (Kanamori et al., 2011; Wu et al., 2014; Bravo-San Pedro et al., 2017), and induced autophagy is needed for further clearance and resistance against acute myocardial ischemic injury and cardiac remodeling (Matsui et al., 2007; Qin et al., 2016).

Post-translational modification (PTM) plays a vital role in protein function after biogenesis. SUMOylation, a reversible PTM, is regarded as a crucial process that controls chromatin structure, gene expression, signal transduction, protein stability, and genome maintenance (Li et al., 2004; Vassileva and Matunis, 2004; Hay, 2005; Guarani et al., 2011; Zhao et al., 2014). During SUMOylation, a small ubiquitin-like modifier (SUMO) protein is covalently attached to substrate proteins *via* an isopeptide bond between its C-terminal glycine and a lysine residue in the substrate protein. The targeted protein is SUMOylated by a cascade of SUMO enzymes E1, E2, and E3. Among these enzymes, E2 conjugation enzyme Ubc9 is known to be the sole E2 enzyme that directly regulates SUMOylation of targeted proteins. Ubc9 is ubiquitously expressed in various cells, governing a pleiotropic cellular pathway. Knockout or downregulation of Ubc9 is detrimental to various organisms, leading to defects in mitosis, chromosome segregation, and other developmental defects (Gupta and Robbins, 2016). Recent studies have reported that overexpression of Ubc9 enzyme activates autophagy, and subsequently improves the cardiac function of desmin-related cardiomyopathy, a proteotoxic disease characterized by the accumulation of protein aggregates and damaged organelles (Gupta et al., 2016).

Therefore, in the present study, we investigated the role of Ubc9 in acute myocardial ischemia. First, we sought to determine whether Ubc9 could be a new target for resisting acute myocardial ischemic injury and ameliorating subsequent myocardial remodeling and cardiac dysfunction. Second, if so, we determined whether autophagy mediated Ubc9’s protective role in acute MI. Third, further determination of the underlying mechanisms, including the details of autophagy regulation and the specific molecules involved, was conducted.

## Methods

### Animal Study Protocol

#### Ethics Statement

The animal study was reviewed and approved by the Institutional Animal Ethics Committee of Guangzhou Medical University (Guangzhou, China). All animal experiments conformed to the National Institutes of Health guidelines (guide for the care and use of laboratory animals)

#### Anesthesia

Adult male C57BL/6 mice were anaesthetized with 1–2% isoflurane and artificially ventilated with a respirator. The efficacy of general anesthesia was assessed by pinching the toe, tail, or ear of the animal. Any reaction from the mouse indicated that the anesthesia was too light and that additional anesthetic agent should be given.

#### Euthanasia

Adult male C57/B6 mice were intraperitoneal injected with an overdose of sodium pentobarbital (100 mg/kg), followed by cervical dislocation. The hearts were collected on days 1, 3, 5, 7, and 14 after MI.

#### MI and Animal Grouping

Mice were subjected to myocardial infarction as described in our previous study (Xiao et al., 2012; Xiao et al., 2015). The sham-operated animals contained an untied left anterior descending artery; that is, suture material was in place, but the ligature was not tightened. Immediately after MI, empty vector adenovirus (carrying GFP, called Adv-GFP; not carrying GFP, called Adv-Null) or Ubc9 adenovirus (carrying GFP or not carrying GFP, uniformly called Adv-Ubc9) were multiple injected into the ischemic zone of the mouse hearts. Mice were randomized into three groups: (i) Sham + Adv-GFP group (or Sham + Adv-Null group), which were sham-operated and multiple injected with Adv-GFP (0.5 × 10^9^ pfu per mouse) or Adv-Null (2.0×10^10^ vp per mouse) (n = 5); (ii) MI + Adv-GFP group (or MI + Adv-Null group), in which MI mice received the same amount of Adv-GFP or Adv-Null (n = 5); and (iii) MI + Adv-Ubc9 group, in which mice were MI-operated and multiple injected with Ubc9 adenovirus carrying GFP (0.5 × 10^9^ pfu per mouse) or Ubc9 adenovirus not carrying GFP (2.0 × 10^10^ vp per mouse) (n = 5). Mice were kept for 5 or 14 days. The success of Ubc9 adenovirus injection was confirmed by GFP fluorescence in frozen slices (**Figure S1A**).

### Antibodies and Reagents

Details are stated in **Supplementary data**.

### Echocardiography

Procedures are stated in **Supplementary data** (Xiao et al., 2012; Xiao et al., 2015).

### TUNEL Staining

The apoptotic cardiomyocytes were stained with TUNEL dye (Gao et al., 2019). Procedures are described in **Supplementary data**.

### Masson’s Trichrome Staining

Fibrosis and percentage of infarct were determined (Xiao et al., 2012). Procedures are stated in **Supplementary data**.

### Cell Culture and Treatment

Neonatal rat cardiomyocytes were used (Gupta et al., 2016). Procedures are stated in **Supplementary data**.

### Transfer With siRNA

We applied siRNA to silence the expression of Ubc9. Procedures are stated in **Supplementary data**.

### Ubc9 Gene Transfer in Cardiomyocytes

Procedures are stated in **Supplementary data**.

### Western Blot Analysis

Procedures are stated in **Supplementary data** (Xiao et al., 2015; Wu et al., 2017).

### Immunoprecipitation and Immunoblotting

Procedures are stated in **Supplementary data** (Zhu et al., 2017).

### Flow Cytometry

The apoptotic cells were analyzed by the Annexin V-APC/7AAD Apoptosis Detection Kit. Procedures are stated in **Supplementary data**.

### Transfection of Adenovirus HBAD-mRFP-GFP-LC3/GFP-LC3

Cells were plated on a confocal plate and infected with adenovirus HBAD-mRFP-GFP-LC3 with 250 (multiplicity of infection, MOI) (Wu et al., 2017). Procedures are stated in **Supplementary data**.

### p62 and Aggresomes Colocalization Assay

Procedures are stated in **Supplementary data**.

### Statistical Analysis

All data are presented as mean ± standard error of the mean. Differences between two groups were analyzed using a Student’s unpaired *t*-test for continuous variables, and using one-way ANOVA when multiple groups were compared. Statistical significance in this study was set at *P < 0.05, **P < 0.01, ***P < 0.001. NS means Not Statistically Significant. All statistical analyses were performed using GraphPad Prism 8.0.1.

## Results

### Ubc9 Improves Cardiac Function and Mitigates Left Ventricular Remodeling After MI

To illustrate the influence of Ubc9 in ischemic heart tissue, we first detected the protein level of Ubc9 after MI. Western blotting showed that Ubc9 expression was significantly elevated from day 3 to day 14 after MI, and the apoptotic marker Cleaved caspase3 was also upregulated not only in the infarct zone but also in the border zone (**Figures S2A, B**).

**Figure 1:**
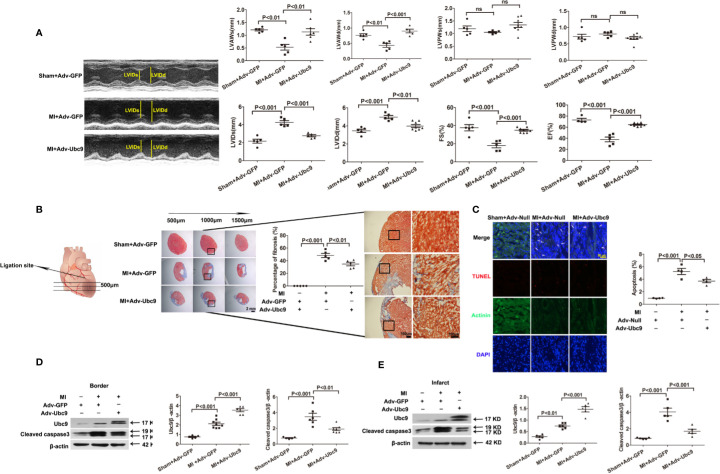
Ubc9 improves cardiac function and alleviates left ventricular remodeling after MI Adv-Ubc9 or Adv-Null was multipoint injected around the ligature of the heart in C57BL/6 mice immediately after MI. **(A)** Representative M-mode images; quantitative analysis of LVIDs, LVIDd, LVAWs, LVAWd, LVPWs, LVPWd, FS, and EF values are presented at day 14 post-MI, n = 5–8 per group. **(B)** Representative images and quantitative analysis of Masson trichrome staining at day 14 post-MI, n = 5 per group. **(C)** Representative images and quantitative analysis of TUNEL-positive cells (red), nuclear cells (DAPI, blue), and cardiomyocytes (α-Actinin, green) at day 5 post-MI; arrows point to apoptotic cells, n = 5 per group. **(D, E)** Western blotting analysis of Ubc9 and Cleaved caspase3 in the border and the infarct area at day 5 post-MI, n = 5–8 per group. Data were analyzed by one-way ANOVA, followed by a Bonferroni post-hoc test.

We then analyzed the effect of Ubc9 overexpression in ischemic heart tissue. Either Adv-Ubc9 or Adv-GFP (or Adv-Null) was multipoint injected into the ischemic zone of heart immediately after MI. First, cardiac function was detected using echocardiography. Adv-Ubc9 mice presented enhanced fractional shortening (FS) and ejection fraction (EF), as well as reduced left ventricular end-systolic dimension (LVIDs) and LV end-diastolic dimension (LVIDd). In addition, Adv-Ubc9 mice also displayed increased LV anterior wall thickness at systole (LVAWs) and diastole (LVAWd), in spite of no marked differences in the LV posterior wall thickness at systole (LVPWs) and diastole (LVPWd) (**Figure 1A**). Next, cardiac remodeling status was analyzed at 14 days post-MI. Masson staining showed that infarct size and myocardial fibrosis were significantly diminished in Adv-Ubc9 mice (**Figure 1B**). Subsequently, the apoptotic changes were assessed at five days post-MI. TUNEL assay showed that Adv-Ubc9 mice had reduced cardiomyocyte apoptosis (**Figure 1C**). Western blotting analysis showed that Cleaved caspase3 was significantly decreased in the infarct and the border zones of Adv-Ubc9 mice (**Figures 1D, E**). All data indicated that Ubc9 overexpression improved cardiac function and alleviated left ventricular remodeling after MI.

### Dynamic Changes in Autophagic Flux After Ubc9 Overexpression Post-MI

Numerous studies have shown that autophagic flux is inadequate at the onset of myocardial ischemia, contributing to instant cardiomyocyte apoptosis and subsequent myocardial fibrosis, and eventual cardiac dysfunction. Therefore, we investigated the change in autophagic flux under Adv-Ubc9 stimulation.

**Figure 2:**
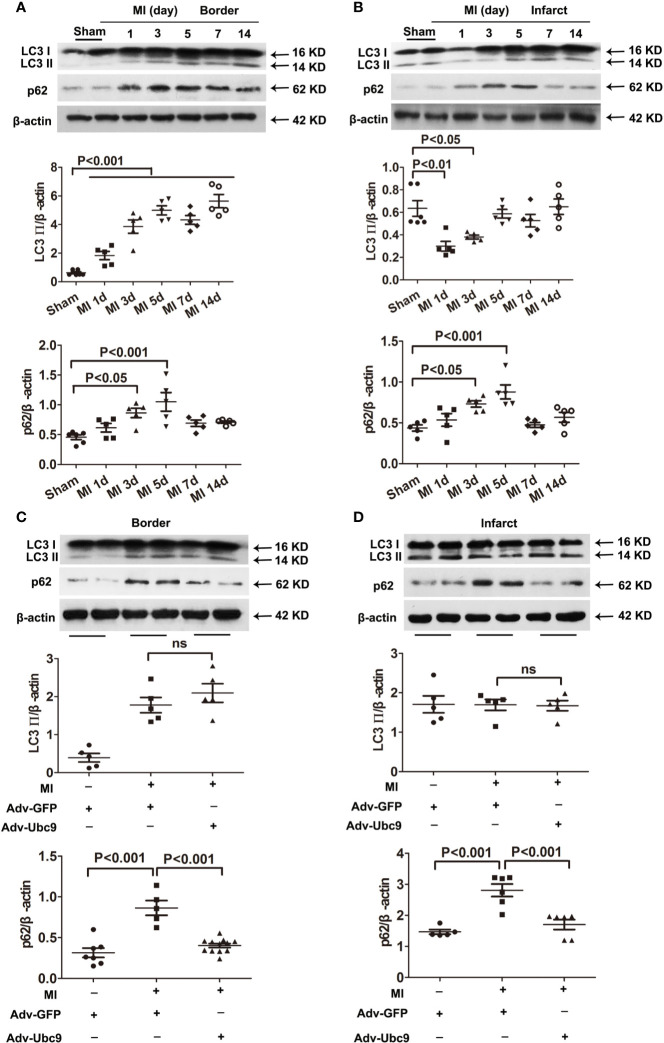
The effects of Ubc9 on autophagic flux in MI **(A, B)** Western blotting analysis of LC3 Ⅱ and p62 in the border and the infarct areas at different time points post-MI, n = 5–6 per group. **(C, D)** Adv-Ubc9 or Adv-GFP was multipoint injected around the ligature of the heart in C57BL/6 mice immediately after MI. Western blotting analysis of LC3 Ⅱ and p62 in the border and the infarct areas at day 5 post-MI, n = 5–7 per group. Data were analyzed by one-way ANOVA, followed by a Bonferroni post-hoc test.

First, we detected the autophagic flux under different MI time points. Western blot assay showed that autophagy marker LC3 II in the border zone was increased on day 1 and sustained increase on days 3−14; another autophagy marker, p62, was increased on days 3 and 5 and decreased on days 7 and 14 (**Figure 2A**). The infarct zone displayed some different results: LC3 II was reduced on days 1 and 3 but recovered to the normal level on day 5 and then remained unchanged on days 7 and 14; p62 showed a similar change to that in the border zone (**Figure 2B**). These results suggest that autophagic flux is insufficient in ischemic myocardium, especially in the acute stage (on days 0−7) of MI.

Second, we performed multiple injections of Adv-Ubc9 into the myocardial ischemic zone and found that accumulation of p62 was reversed in the infarct and the border areas post-MI. Intriguingly, LC3 II was unchanged in these two areas (**Figures 2C, D**). These data suggest that Ubc9 overexpression significantly promotes autophagic flux in myocardial ischemia by enhancing p62 clearance.

### Ubc9 Enhances Cardiomyocyte Survival During OGD Stress

**Figure 3:**
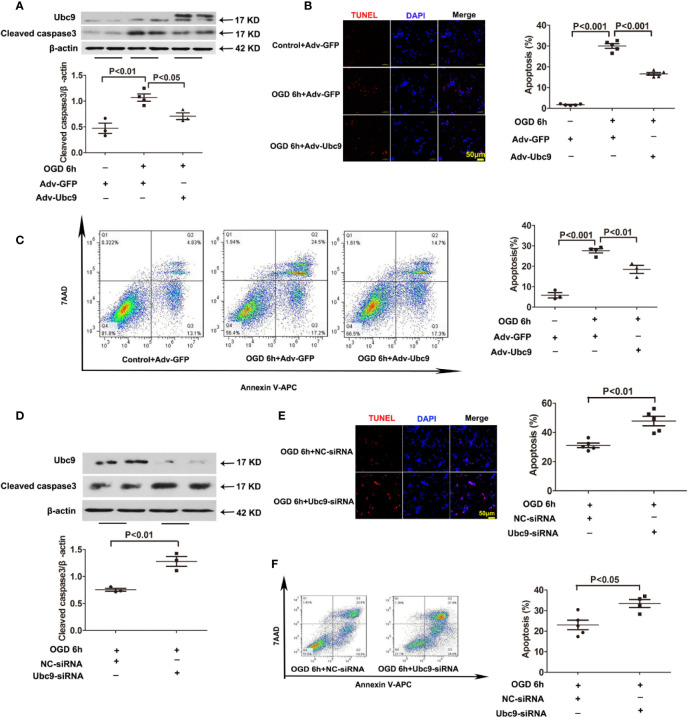
Ubc9 overexpression suppresses OGD-induced cardiomyocyte damage, and Ubc9 silencing exacerbates cardiomyocyte apoptosis under OGD treatment NRCMs were treated with Adv-Ubc9 or Adv-GFP for 12 h, and then cultured for another 36 h in fresh DMEM. After that, OGD was applied for 6 h. Alternately, short interfering RNA targeting Ubc9 or negative control siRNA was transfected in NRCMs after 48 h; the cells were in hypoxia status for 6 h. **(A, D)** Western blotting analysis of Ubc9 and Cleaved caspase3, n = 3–4 per group. **(B, E)** Representative images and quantitative analysis of TUNEL-positive cells (red) and cell nucleus (DAPI, blue), n = 5 per group. **(C, F)** Representative images and quantitative analysis of flow cytometry, n = 3–5 per group. Data were analyzed by one-way ANOVA, followed by a Bonferroni post-hoc test, or analyzed by unpaired Student’s t test.

To better explore the influence of Ubc9 in myocardial ischemia, we investigated the role of Ubc9 in cardiomyocytes *in vitro*. First, we detected the protein levels of Ubc9, Cleaved caspase3, and autophagy markers LC3 II and p62 after OGD. The western blotting assay showed that Cleaved caspase3 was significantly upregulated 4 and 6 h after OGD, and p62 was upregulated 6 and 12 h after OGD, but LC3 II was downregulated at the onset of OGD and sustained this status during the entire OGD stress period (**Figures S3A, B**). Then, we transfected Adv-Ubc9 or Ubc9-siRNA in NRCMs before OGD stimulation. Overexpression of Ubc9 significantly decreased the protein level of Cleaved caspase3 under OGD conditions (**Figure 3A**), and it also decreased cardiomyocyte apoptosis under OGD treatment as evidenced by TUNEL assay (**Figure 3B**) and flow cytometry (**Figure 3C**). However, Ubc9-siRNA further increased the level of Cleaved caspase3 under OGD treatment (**Figure 3D**) and led to more cardiomyocyte apoptosis than negative control siRNA (NC-siRNA) after OGD stimulation, as shown by TUNEL assay (**Figure 3E**) and flow cytometry analysis (**Figure 3F**). These results indicate that Ubc9 indeed protects cardiomyocytes from OGD-induced damage.

### Ubc9 Ameliorates Autophagic Flux Disorder Under OGD Through p62 Clearance Enhancement

**Figure 4:**
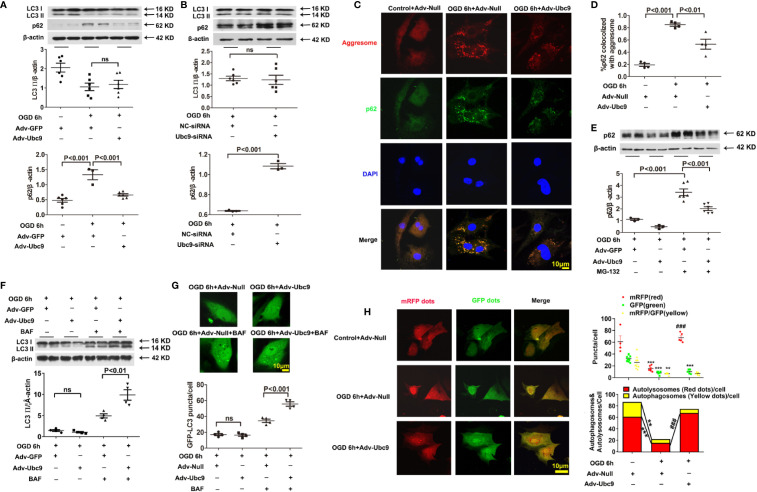
Ubc9 ameliorates the impaired autophagic flux through increasing autophagy formation and autophagy degradation NRCMs were treated with Adv-Ubc9 (or Adv-Null) or Ubc9-siRNA (or NC-siRNA) for 48 h. After that, OGD was applied for 6 h. **(A, B)** Western blotting analysis of LC3 Ⅱ and p62, n = 3–6 per group. ** (C, D)** Representative images and quantitative analysis of p62 colocalized with aggresomes. Aggresomes were visualized with an aggresome detection kit (red), and p62 was visualized by immunostaining with antibody (green), n = 4 per group. **(E)** NRCMs were treated with Adv-Ubc9 or Adv-GFP for 24 h, and then co-treated with MG-132 (1 μM) for another 24 h. After that, OGD was applied for 6 h, co-treating with MG-132 (1 μM). Western blotting analysis of p62, n = 3–6 per group. **(F)** NRCMs were treated with Adv-Ubc9 or Adv-GFP for 46 h, and then co-treated with BAF (50 nM) for another 2 h. After that, OGD was applied for 6 h, co-treating with BAF (50 nM). Western blotting analysis of LC3 Ⅱ, n = 4 per group. **(G)** After Adv-Ubc9 or Adv-Null was transfected for 12 h, Adv-GFP-LC3 or Adv-Null was co-transfected for another 34 h. Next, BAF (50 nM) was applied for another 2 h. After that, OGD was applied for 6 h, co-treating with BAF (50 nM). Representative images and number statistics of GFP-LC3 puncta (green) per cell, n = 4 per group. **(H)** NRCMs were treated with Adv-Ubc9 or Adv-Null for 12 h, and then co-transfected with Adv-mRFP-GFP-LC3 for another 48 h. After that, OGD was applied for 6 h. Representative images and number statistics of GFP dots (green), mRFP dots (red), autophagosomes, and autolysosomes per cell, **P < 0.01, ***P < 0.001 vs. the controls; ^###^P < 0.001 vs. the OGD 6h+Adv-Null group, n = 4–9 per group. Data were analyzed by one-way ANOVA, followed by a Bonferroni post-hoc test, or analyzed by unpaired Student’s t test.

To further investigate the action of Ubc9 in autophagic flux under myocardial ischemia, we detected the levels of LC3 II and p62 in cardiomyocytes after treatment with Adv-Ubc9 or Ubc9-siRNA under OGD status. The results showed that the accumulation of p62 was significantly alleviated, but the LC3 II was still unchanged after delivery of Adv-Ubc9, similar to the results of the *in vivo* experiments (**Figure 4A**). However, the accumulation level of p62 was increased, and LC3 II was still unchanged after Ubc9-siRNA treatment (**Figure 4B**), suggesting that Ubc9 improves the impaired autophagic flow in cardiomyocytes undergoing OGD through enhancing the p62 clearance.

P62 has two different roles in degradation. It can act as a “cargo” protein that connects LC3 II with the impaired proteins and organelles, which form as aggresomes that are degraded by autolysosome. Therefore, the accumulation of p62 and aggresomes were used to analyze autophagic clearance. Data showed that colocalization of p62 and aggresomes were significantly accumulated during OGD conditions, and Ubc9 delivery reduced this accumulation (**Figures 4C, D**), suggesting enhanced protein clearance in autophagic flux. However, p62 could also conjugate with ubiquitinated protein and be degraded by the ubiquitin-proteasome system (UPS) (Chondrogianni et al., 2014). The previous study showed that additional Ubc9 level s could help to regulate UPS function (Gupta et al., 2014). Therefore, we inhibited the ubiquitin-proteasomal degradation of NRCMs with MG-132 (1 µM) treatment 24 h before OGD. The western blotting assay showed that the Adv-Ubc9 group still performed a significant reduction in the p62 level compared with the GFP group in the presence of MG-132 (**Figure 4E**), implying that Ubc9 plays a separate role in the autophagic clearance, independent of ubiquitin-proteasome system degradation under OGD stress.

### Activation of Autophagy Formation and Autophagic Degradation Mediates the Benefits Brought by Adv-Ubc9 Under OGD

It is intriguing that LC3 II level was hardly changed after Ubc9 stimulation in our *in vivo* and *in vitro* experiments. LC3 II level is related to autophagosome formation and autophagic degradation. Therefore, we applied further experiments to determine the autophagy status augmented by Ubc9.

First, the autophagy inhibitor BAF (50 nM) was delivered for 2 h before OGD. The results showed that Adv-Ubc9 promoted an increase in LC3 II level compared with Adv-GFP in the presence of BAF, which is different the change that occurred in the absence of BAF (**Figure 4F**). Meanwhile, GFP-LC3 fluorescence assay also confirmed this finding, as indicated by augmented numbers of green puncta in Adv-Ubc9, in the presence of BAF (**Figure 4G**). These results suggest that autophagosome formation is activated unambiguously by Ubc9.

We then monitored autophagic flux using mRFP-GFP tandemly tagged LC3 (mRFP-GFP-LC3). The GFP signal is quenched inside lysosomes; thus, autophagosomes and their precursors are marked by both GFP and RFP (yellow puncta), whereas autolysosomes are labeled with RFP (red puncta). More specifically, yellow plus red puncta refers to the formation of the whole autophagosome, and the ratio of red to yellow puncta refers to the capability of autophagosome-lysosome fusion or autophagic degradation that is independent of upstream autophagosome numbers (Kimura et al., 2007). Compared with Adv-Null, Adv-Ubc9 largely increased the numbers of yellow plus red puncta as well as the ratio of red to yellow puncta. Moreover, this process occurred more in the form of autolysosomes, as measured by the largely increased red puncta (**Figure 4H**). These results suggest that Ubc9 enhances autophagy by activating both the autophagosome formation and the autophagic degradation processes under OGD conditions, simultaneously.

**Figure 5:**
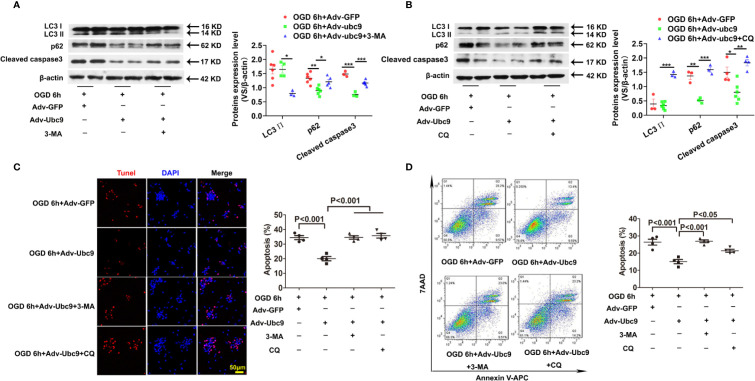
Energetic autophagy flux mediates the protective effects of Ubc9 on cardiomyocytes under OGD Adv-Ubc9 or Adv-GFP was transfected in NRCMs for 36 h, and then co-treated with 3-MA (5 mM) or CQ (20 μM) for another 12 h. After that, OGD was applied for 6 h, co-treating with 3-MA (5 mM) or CQ (20 μM) for 6 h. **(A, B)** Western blotting analysis of LC3 Ⅱ, p62, and Cleaved caspase3, *P < 0.05, **P < 0.01, ***P < 0.001 versus corresponding group, n = 3–7 per group. **(C)** Representative images and quantitative analysis of TUNEL-positive cells (red) and cell nucleus (DAPI, blue), n = 4 per group. **(D)** Representative images and quantitative analysis of flow cytometry, n = 4 per group. Data were analyzed by one-way ANOVA, followed by a Bonferroni post-hoc test.

To determine if the protective role of Ubc9 was mediated by autophagic flux enhancement under OGD, either the autophagosome formation or the autophagic degradation was inhibited through pretreatment with 5 mM 3-MA (3-Methyladenine) or 20 μM CQ (Chloroquine) for 12 h before OGD, respectively. Compared with the Adv-Ubc9 group, the Adv-Ubc9 plus 3-MA group presented decreased LC3 II and increased p62 (**Figure 5A**), and the Adv-Ubc9 plus CQ group presented increased LC3 II and reduced p62 under OGD stimulation (**Figure 5B**). We then detected cardiomyocyte apoptosis under such treatments. Compared with the Adv-Ubc9 group, the Adv-Ubc9 plus 3-MA and CQ groups both showed an enhanced level of Cleaved caspase3 (**Figures 5A, B**) and numbers of TUNEL-positive cells (**Figure 5C**). Flow cytometry assay presented similar results to the TUNEL staining (**Figure 5D**).

In addition, OGD-induced cardiomyocytes were treated with 3-MA or CQ, as a control of Adv-Ubc9 plus 3-MA or CQ under OGD status. Data showed that 3-MA and CQ significantly inhibited autophagy during OGD stimulation. Moreover, CQ increased apoptosis while 3-MA presented little effect on apoptosis, as indicated by Cleaved caspase3 level. Owning to the apoptosis induction in this dose of CQ, the result in Adv-Ubc9 plus CQ group may include the proapoptotic performance of CQ itself, which indicates that lower dose of CQ is more suitable (**Figure S4A**). Moreover, 3-MA has been reported to induce autophagy in some conditions (Wu et al., 2010), and then we applied BAF to prevent autophagosome-lysosome fusion in the 3-MA group to confirm its real role in our present study. Data showed that after BAF impeded the autophagy flux, 3-MA still significantly inhibited autophagy, as indicated by LC3 II level (**Figure S4B**).

All the above results indicate that both autophagosome formation and autophagic degradation mediate the protection of Ubc9 to OGD-induced cardiomyocytes.

### Ubc9 Has No Significant Impact on the Classic Upstream Molecules of Autophagosome Formation

Next, we investigated the autophagy molecular target of Ubc9 under ischemia. We first focused on the classic molecules related to autophagosome formation. The western blotting assay showed that Ubc9 had no significant effect on these proteins (p-AMPK, AMPK, Beclin1, p-mTOR, mTOR, p-ULK555, p-ULK757, and ULK) *in vitro* (**Figures S5A, B**), suggesting the non-targeting of Ubc9 to these pathways.

### Ubc9 Increases Cardiac SUMOYlated Protein Levels Under Ischemia

Next, we focused on whether the overexpression of Ubc9 affected SUMOylation level of autophagy proteins under ischemia. However, before that, we needed to detect the level of globally SUMO-conjugated proteins under Ubc9 stimulation. Until now, three isoforms of SUMO have been found in mammalian cells: SUMO-1, SUMO-2, and SUMO-3. Because SUMO-2 and SUMO-3 are nearly 97% identical under most contexts, they are jointly regarded as SUMO-2/3. Contrarily, SUMO-1 is only about 47% identical to SUMO-2/3 (Saitoh and Hinchey, 2000). In fact, SUMO-1 and SUMO-2/3 each have a specific pool of targets while sharing some overlay in protein modification, indicating that they may perform differently in cellular processes (Shimizu et al., 2016). The western blotting assay from the present study showed no significant changes of global SUMO-1 and SUMO-2/3 conjugated proteins *in vivo* (**Figures S6A, B**) and *in vitro* (**Figures S6E, F**) at different time points under stress. After overexpression of Ubc9, the total SUMO-1 and SUMO-2/3 conjugated protein levels were significantly increased *in vivo* (**Figures S6C, D**) and *in vitro* (**Figures S6G, H**), indicating that Ubc9 increases cardiac SUMOylation level under ischemia.

### Ubc9 Boosts PI3K-III Complex Assembly and Autophagy-Positioning

**Figure 6:**
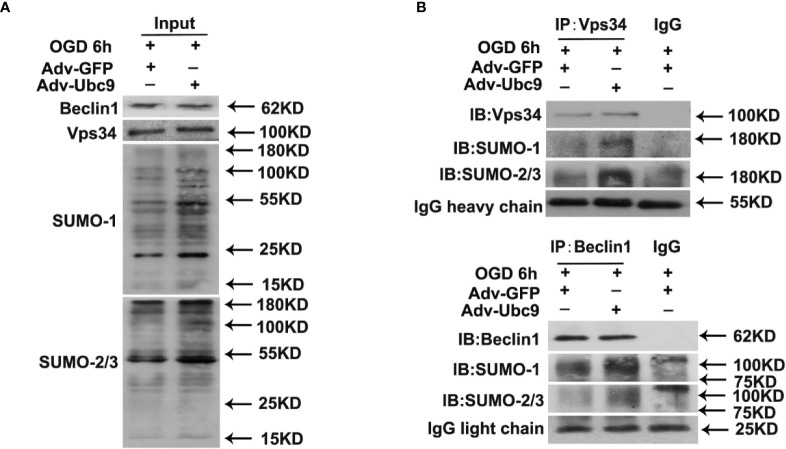
Ubc9 increases the SUMOylation of Beclin1 under OGD treatment Adv-Ubc9 was transfected in NRCMs for 48 h, then maintained in hypoxic condition for 6 h. **(A, B)** Endogenous SUMOylation of Vps34 and Beclin1 in NRCMs. Beclin1 or Vps34 was IP, and followed by IB for SUMO-1 or SUMO-2/3.

We then detected the autophagy proteins modified by SUMO under Ubc9 stimulation. The SUMOsp 2.0 software was applied to predict SUMO-modified site numbers of the autophagy proteins (**Table S1**). From the table, we found that PI3K-III complex components Vps34, Vps15, and Beclin1 had relatively more SUMOylation sites than others. Among them, Vps34 and Beclin1 are the core proteins in the PI3K-III complex, which are essential for complex assembly and activity (Backer, 2016). Therefore, we immunoprecipitated Vps34 or Beclin1 from the cell lysates of Ubc9 stimulation, and detected SUMOylation. Data showed that not only Vps34 but also Beclin1 displayed increased SUMO1 and SUMO2/3 after Ubc9 stimulation (**Figures 6A, B**).

The PI3K-III complex is essential for autophagic flux, participating in autophagosome formation and autophagosome-lysosome fusion (Backer, 2016). It often exists as two patterns, PI3K-III complex I and II. The recruitment of the Vps15-Vps34-Beclin1-ATG14 complex (also named PI3K-III complex (I) to autophagosome is necessary for autophagosome formation; The recruitment of the Vps15-Vps34-Beclin1-UVRAG complex to autolysosome (also named PI3K-III complex (II) is responsible for autophagic degradation (Backer, 2016; Bento et al., 2016; Cheng et al., 2017).

**Figure 7:**
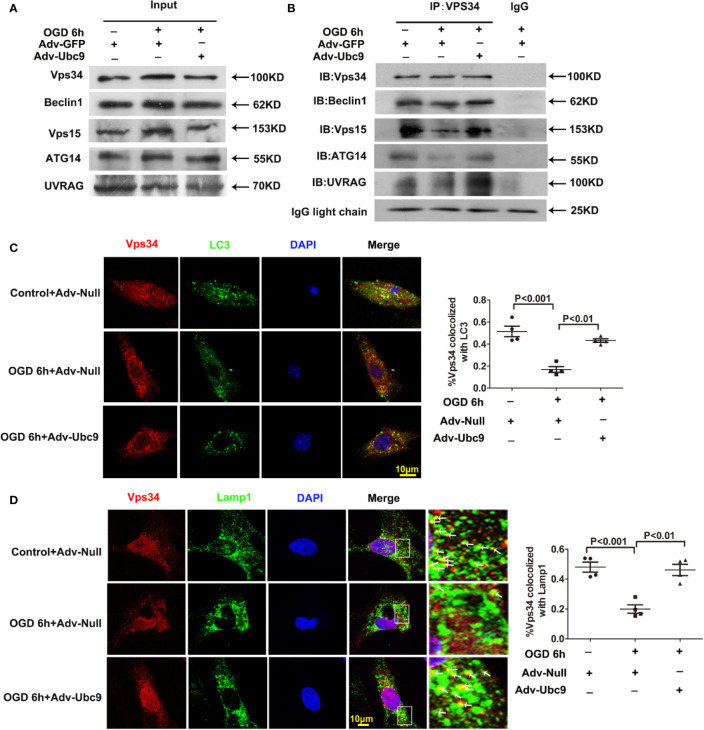
Ubc9 promotes the formation and the autophagy-positioning of PI3K-III complexes Adv-Ubc9 was transfected in NRCMs for 48 h, then maintained in hypoxic condition for 6 h. **(A, B)** Endogenous PI3K-III complexes in NRCMs. Vps34 was IP, and followed by IB for Beclin1, Vps15, ATG14, and UVRAG. **(C)** Fixed cells were immunostained with antibodies of Vps34 (red) and LC3 (green), and confocal microscopy for colocalization to percentage of Vps34 and LC3, n = 4 per group. **(D)** Fixed cells were immune-stained with antibodies of Vps34 (red) and Lamp1 (green), confocal microscopy for colocalization to percentage of Lamp1 and Vps34. Magnifications of the boxed area are shown in the right; arrows point to colocalization sites of Vps34 with Lamp1, n = 4 per group. Data were analyzed by one-way ANOVA, followed by a Bonferroni post-hoc test.

Therefore, we detected the complex assembly and autophagy orientation after Ubc9 overexpression. Little change was presented in the basal protein levels of Beclin1, Vps34, Vps15, ATG14, and UVRAG by immunoblotting analysis. However, applying Vps34 antibody to pull down Beclin1, Vps15, ATG14, and UVRAG showed that Adv-Ubc9 increased the binding of Vps34-Beclin1, Vps34-Vps15, Vps34-ATG14, and Vps34-UVRAG, indicating the enhancement of PI3K-III complex formation (**Figures 7A, B**). Our data also showed that Adv-Ubc9 significantly increased the colocalization of Vps34 with LC3 and Vps34 with Lamp1 (**Figures 7C, D**). All the results above imply that boosting complex assembly and autophagy-orientation through Ubc9 overexpression may participate in Ubc9-induced autophagic flux enhancement, which might be related to Vps34-Beclin1 SUMOylation.

## Discussion

In the present study, we investigated the effects of SUMO E2 conjugating enzyme Ubc9 on cardiac injury after acute MI. Our significant findings in this study were (i) Ubc9 overexpression reduces cardiac damage and preserves cardiac function after acute MI, (ii) enhancement of autophagic flux from activation to degradation mediates the protective role of Ubc9, and (iii) promoting the formation of PI3K-III complexes and autophagy-positioning by Ubc9 overexpression may facilitate this autophagy regulation.

### Ubc9, a Potential Target in Myocardial Ischemic Disease

The current standard of care for patients with acute coronary occlusion is reperfusion therapy. Primary percutaneous coronary intervention improves the circulation of epicardial coronary system; however, the endocardium is persistently hypo-perfused in a substantial portion of patients (De et al., 2017). This phenomenon, called no-reflow, results from severe microvascular dysfunction or loss of integrity leading to microvascular obstruction (MVO). MVO, plus other coronary artery diseases, lead to instant myocardial tissue damage, subsequent cardiac remodeling, long-term heart failure, and even mortality. In this series of processes, the fastest emerging and dominant performances are the direct destruction of cardiomyocytes in the ischemic zone and the indirect injury to cardiomyocytes in the border zone. Therefore, the protection of cardiomyocytes is vital for management of acute ischemic heart diseases.

Ubc9 can function as a transcriptional co-regulator as well as a SUMO E2 ligase. In this study, we report that myocardial injection of adenovirus Ubc9 triggers increased Ubc9 expression and SUMOylation levels, which lead to decreased cardiomyocyte apoptosis in the infarct and peri-infarct zones. Importantly, this resistance to apoptosis results in reduced infarct ratio and peri-infarct fibrosis, and subsequently improves cardiac function.

The *in vitro* experiments in our study further confirm the protective role of Ubc9 for cardiomyocytes. Our data from Ubc9 overexpression and Ubc9 silencing tests indicate that endogenous Ubc9 is beneficial to ischemic cardiomyocytes, and when exogenous Ubc9 is applied, the apoptosis of the hypoxic cardiomyocytes is significantly reversed, which is critical at the beginning of the repair process after acute myocardial infarction.

We are not the first to investigate the role of SUMO-modification in ischemic heart diseases. One previous study about SUMO-1 gene therapy demonstrated that SUMO-1 modification of cardiac SERCA2a increases its activity and improves cardiac contractility in heart failure (Changwon et al., 2012; Tilemann et al., 2013). Similar to this previous study, our results also suggest that SUMO-modification can cause improvements in cardiac function. However, there are fundamental differences in our investigation that set this work apart from previously published studies. First, we established a myocardial infarction model using permanent ligation of coronary arteries, while other studies used 2-h balloon occlusion of the proximal or mid-left anterior descending coronary artery followed by reperfusion to induce myocardial injury. Second, we used SUMO E2 conjugating enzyme Ubc9, but not SUMO-1 itself. Ubc9 regulates the modification related to not only SUMO-1 but also SUMO-2/3. Third, we delivered the adenovirus immediately after the ligation of a coronary artery, rather than one month after MI, which means that we focused on instant protection at the onset of acute cardiomyocyte injury.

### Ubc9 and Autophagic Flux Regulation

Autophagy is a conserved intracellular degradation pathway that envelopes substrates, such as bulk cytoplasm, organelles, aggregate-prone proteins, and infectious agents, into double-membrane vesicles, and then transports them to lysosomes to degrade the “trash” and release useful elements (Bento et al., 2016). This process facilitates cell restoration against starvation and related stresses among many diseases and physiological situations (Lavandero et al., 2015; Rubinsztein et al., 2015).

During various pathological conditions of the heart, including pressure overload, ischemia/reperfusion (I/R), and MI, myocardial autophagy can be adaptively activated. This compensatory activation is able to mitigate energy loss and clean damaged mitochondria and protein aggregates (Yan et al., 2005; Hongxin et al., 2007). Taking acute myocardial infarction as an example, most previous studies report that autophagy is activated in the border zone of acute MI (Chiang et al., 2017; Wu et al., 2017); however, the impaired proteins and organelles, formed as aggresomes, and/or the autophagic degradation-related protein p62 are still largely accumulated, suggesting insufficient clearance by autophagy. This is consistent with our finding that autophagy marker LC3 II, together with upstream factors (AMPK, Beclin1, ULK, etc.) of autophagosome formation, is upregulated, but the protein expression of p62 is accumulated in the MI border zone. Our results also suggest that autophagosome formation is downregulated in the infarct zone, as indicated by the decreased LC3 II and the increased p62 proteins, which we also confirmed *in vitro*, as indicated by the downregulated autophagosome formation that occurred in the neonatal cardiomyocytes under the fierce deprivation of oxygen, glucose, and serum. The expressions of LC3 II and p62 in the infarct zone later returned to an approximately normal level, which might result from the gradual increase of cardiac fibroblasts entering into this ischemic zone. Therefore, enhancement of autophagic flux is indispensable for rescuing the myocardium not only in the border zone but also in the infarct zone of acute MI.

Autophagic flux has two integral parts, including autophagosome formation and autophagic degradation. Autophagosome formation is a process of forming double-membrane organelles enveloping excessive or aberrant organelles and protein aggregates, whereas autophagic degradation delivers the autophagosomes to the lysosomes for clearance and recycling. Multiple studies have stated that the acceleration of autophagic flux is protective under ischemic stress (Matsui et al., 2007; Sciarretta et al., 2018). Among these studies, most of them enhance autophagic flux by boosting autophagosome formation, and few studies promote autophagic flux through increasing autophagic degradation. Our research present that Ubc9 reduces the protein level of p62 and the fluorescence of colocalization for p62 and aggresomes, while the LC3 II protein level is barely changed. After applying BAF to curb autophagosome-lysosome fusion, LC3 II expression in Ubc9 delivery is efficiently elevated. This is consistent with the mRFP-GFP-LC3 fluorescence assay results (i.e., after overexpression of Ubc9), in which the red puncta are increased, and the yellow puncta are nearly still, but the total quantity of red and yellow puncta and the ratio of red to yellow puncta are all increased, indicating that Ubc9 facilitates autophagic flux through simultaneously regulating autophagy formation and autophagic degradation.

Although Ubc9 indeed influences the autophagic flux from formation to degradation, we need to apply autophagy inhibitor to confirm the autophagic role of Ubc9 on cardiomyocytes survival. 3-MA and CQ were used respectively to impede the autophagosome formation and the autolysosome degradation, which effectively reverses the role of Ubc9 on autophagy and significantly counteracts the protection of Ubc9 on cardiomyocytes under OGD.

Indeed, in recent years, there have been increasing numbers of studies unveiling new information on autophagy. Emerging evidence has revealed that autophagy may also promote cell death through excessive autophagosomes formation, which can be obtained from recent papers (Wang et al., 2015; Gao et al., 2018; Gao et al., 2019). These groups demonstrate that inhibition of redundant autophagosomes effectively protects cardiomyocytes from ischemic injury. Therefore, accelerating autophagy formation and autophagic degradation at the same time (e.g., in our present study) is better than manipulating only one of them, and the former can efficiently boost autophagic flux and avoid excessive autophagosomes.

In addition, our results partly differ from those of another investigation (Gupta et al., 2016) that studied the role of Ubc9 in autophagy, focusing exclusively on autophagosome formation. Although the investigation by Manish K. Gupta et al. (Gupta et al., 2016) and ours have the same results for the tandem fluorescent-tagged LC3 (tfLC3) assay, the expression of LC3 II and p62 are different. The discrepancy may be explained as follows. (1) The differences in the background of the animal model. The disease in our research is ischemic heart disease, and the stimulation used *in vitro* is OGD, whereas their study focuses on proteotoxic disease, and the *in vitro* experiment employs normal cells and/or starved cells. (2) LC3 II level is determined by autophagosome formation and autophagic degradation, while p62 level is affected by autophagy and ubiquitination; hence, the changing status may lead to changing results.

### Ubc9, PI3K-III Complexes, and Autophagy

We then chose autophagy molecules that are rich in SUMO sites through SUMOsp 2.0 software. From these molecules, we detected the upregulation of SUMOylation in Vps34 and Beclin1 by CO-IP, which are critical molecules in PI3K-III complexes. This finding warrants further investigation into the formation of PI3K-III complexes.

PI3K-III complex I includes Vps34, Beclin1 and ATG14, involved in autophagosome formation; PI3K-III complex II contains Vps34, Beclin1 and UVRAG, engaged in autophagosome-autolysosome fusion. From CO-IP assay, we found increased PI3K-III complexes, as indicated by elevated interactions in Vps34-Bbeclin1, Vps34-ATG14, and Vps34-UV RAG, implying increased PI3K-III complex I (Vps34-Beclin1-ATG14) and complex II (Vps34-Beclin1-UVRAG) under Adv-Ubc9 stimulation. We focused on not only the formation of PI3K-III complex I and II but also their status on autophagy. We found that Ubc9 increased colocalization of Vps34 with autophagosome marker LC3 and colocalization of Vps34 with autolysosome marker Lamp1, indicating that increased PI3K-III complexes participate in autophagosome formation and autolysosome degradation.

This is the first investigation to identify Ubc9 as a target that facilitates autophagic flux from autophagosome formation to autophagic degradation through the common molecule PI3K-III complexes.

The above mechanisms also raise several intriguing questions. First, although we have confirmed that PI3K-III complexes are involved in Ubc9-induced autophagy enhancement, the regulation details of Vps34-Beclin1 SUMOylation in this process are still unclear. Second, we have chosen the candidate molecules only using SUMOsp 2.0 software, which may have missed some targets possessing less but effective SUMOylation sites, or other targets that are mistaken as having fewer SUMOylation sites (Flotho and Melchior, 2013). Third, autophagic degradation, which makes up a large proportion of Ubc9-induced autophagic flux enhancement in our present study, should be further investigated.

## Conclusion

Our present findings discover that SUMO E2 conjugating enzyme Ubc9 upregulates autophagic flux to resist cardiac injury immediately after ischemic stress in the heart. Notably, the formation of PI3K-III complexes and autophagy-positioning are involved in Ubc9-induced autophagic flux enhancement, which might be related to the SUMOylation of Vps34-Beclin1. Identification of this target will enhance the development of clinical therapies for ischemic heart diseases.

## Data Availability Statement

All datasets presented in this study are included in the article/**Supplementary Material**.

## Ethics Statement

The animal study was reviewed and approved by The Institutional Animal Ethics Committee of Guangzhou Medical University.

## Author Contributions

QX contributed to conception and design, provision of study material, collection and/or assembly of data, data analysis and interpretation, manuscript writing, and final approval of the manuscript. X-HC, R-CJ, S-YC, K-FC, XZ, X-LZ, and J-JH contributed to collection and/or assembly of data and final approval of the manuscript. YQ and G-PZ contributed to provision of study material and final approval of the manuscript. J-DL and QY contributed to conception and design, financial support, administrative support, provision of study material, data analysis and interpretation, manuscript writing, and final approval of manuscript.

## Funding

This study was supported by the National Natural Science Foundation of China (grant no. 81302767 to QX and grant no. 81573433 to J-DL), the Science and Technology Planning Project of Guangdong Province (grant no. 2017A020215194 to QX), the Medical Scientific Research Foundation of Guangdong Province (grant no. B2018054 to Xiang Zhu), and the PhD Start-up Fund of Natural Science Foundation of Guangdong Province (grant no. 2017A030310458 to J-JH.

## Conflict of Interest

The authors declare that the research was conducted in the absence of any commercial or financial relationships that could be construed as a potential conflict of interest.
