# Supplementary material for: Ubc9 Attenuates Myocardial Ischemic Injury Through Accelerating Autophagic Flux
Source: Front Pharmacol. 2020 Sep 15;11:561306. doi: 10.3389/fphar.2020.561306 (PMC7522513; doi:10.3389/fphar.2020.561306)
Supplement: Supplementary file 1 [file Presentation_1.pdf]

Ubc9 attenuates myocardial ischemia injury through accelerating autophagic flux

Qing Xiao[a,b,c,1], Xiu-Hui Chen[b,1], Ru-Chao Jiang[b], Sheng-Ying Chen[b], Kai-Feng Chen[b], Xiang Zhu[b], Xiao-ling Zhang[d], Jun-jun Huang[a], Yuan Qin[a,b], Gui-ping Zhang[a,b], Quan Yi[a,b,*], Jian-dong Luo[a,b,c,*]

[a]Key Laboratory of Molecular Target & Clinical Pharmacology, School of Pharmaceutical Sciences & the Fifth Affiliated Hospital, Guangzhou Medical University, Guangzhou, Guangdong 511436, P.R. China;
[b]Department of Pharmacology, Guangzhou Medical University, Guangzhou, Guangdong 511436, P.R. China;
[c]Guangzhou Institute of Cardiovascular Disease, Guangzhou Key Laboratory of Cardiovascular Disease, The Second Affiliated Hospital, Guangzhou Medical University, Guangzhou, Guangdong 511436, P.R. China;
[d]Maternal and Children Hospital of Guangdong Province, Guangzhou, Guangdong 510260, P.R. China

Short title: Ubc9 regulates autophagic flux during MI.

[1]Qing Xiao and Xiu-hui Chen contributed equally to this work.

Correspondence to
Professor Jian-dong Luo, M.D.,
Email: jiandongluo@hotmail.com
Or Professor Quan Yi, M.D.,
Email: yiquan@gzhmu.edu.cn
Department of Pharmacology, Guangzhou Medical University, Xinzao, Panyu District, Guangzhou, 511436, P.R. China
Tel: +86-20-37103000
Fax: +86-20-37103099

Supplementary Figures and Tables

Figure S1

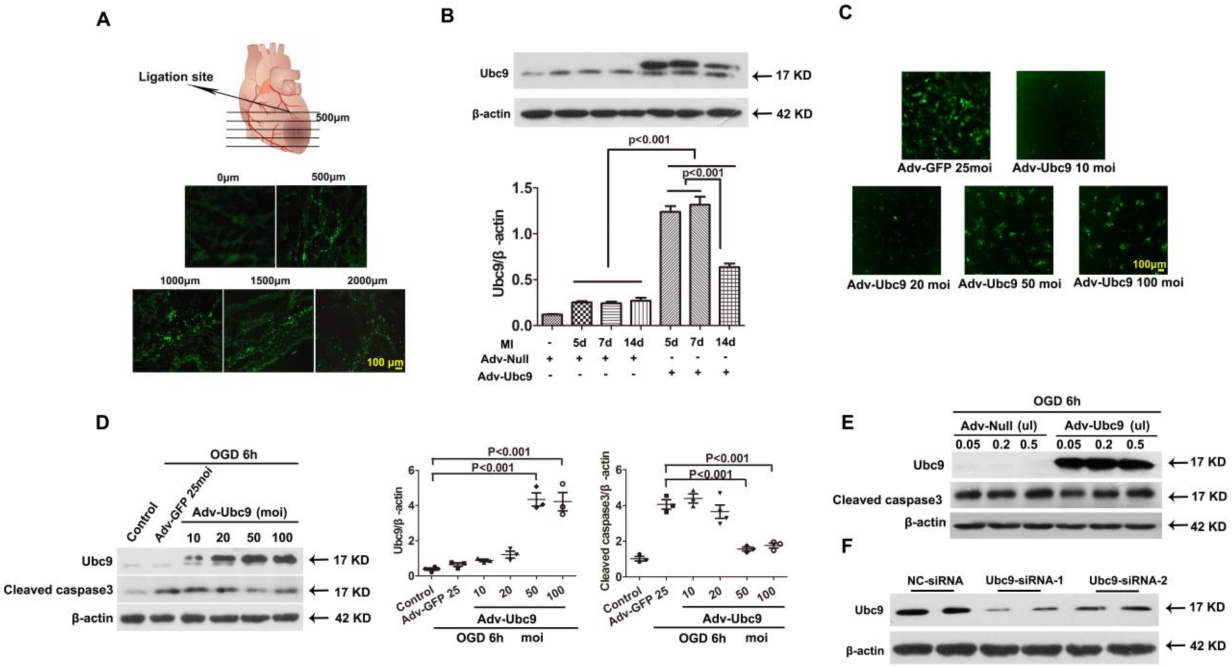

Figure S1. Transfer efficiency assessments of Ubc9 overexpression and silence

(A) Ubc9 adenovirus carrying GFP was multiple injected in the heart (0.5×10^9 pfu per heart), the heart was removed at day 5 post-MI. Frozen sections were prepared. Slices position of heart pattern, and the fluorescence intensity of GFP was observed under a fluorescence microscope and photographed, the photographs were arranged from the ligation point to the apical region. (*B*) The hearts were multiple injected by Ubc9 adenovirus, and then removed at day 5, 7 and 14 post-MI. Western blotting analysis of Ubc9, n=4-5 per group. (*C*, *D*) NRCMs were infected with Adv-GFP 25 moi, and Adv-Ubc9 transfected with 10, 20, 50 and 100 moi for 48 h, respectively, and then maintained in hypoxic condition for 6 h. The fluorescence intensity of GFP was observed under fluorescence microscope and photographed; Western blotting analysis of Ubc9 and Cleaved caspase3, n=3-4 per group. (*E*) Divided Adv-Null or Adv-Ubc9 into three volumes: 0.05ul, 0.2ul, 0.5ul, and transferred them in NRCMs, culturing with 1 ml DMEM added 10% FBS for 48 h and then maintained in hypoxic condition for 6 h. Western blotting analysis of Ubc9 and Cleaved caspase3. (*F*) Ubc9-siRNA-1 and Ubc9-siRNA-2 were transfected in NRCMs 48 h. Western blotting analysis of Ubc9. Data were analyzed by one-way ANOVA, followed by a Bonferroni post-hoc test.

Figure S2

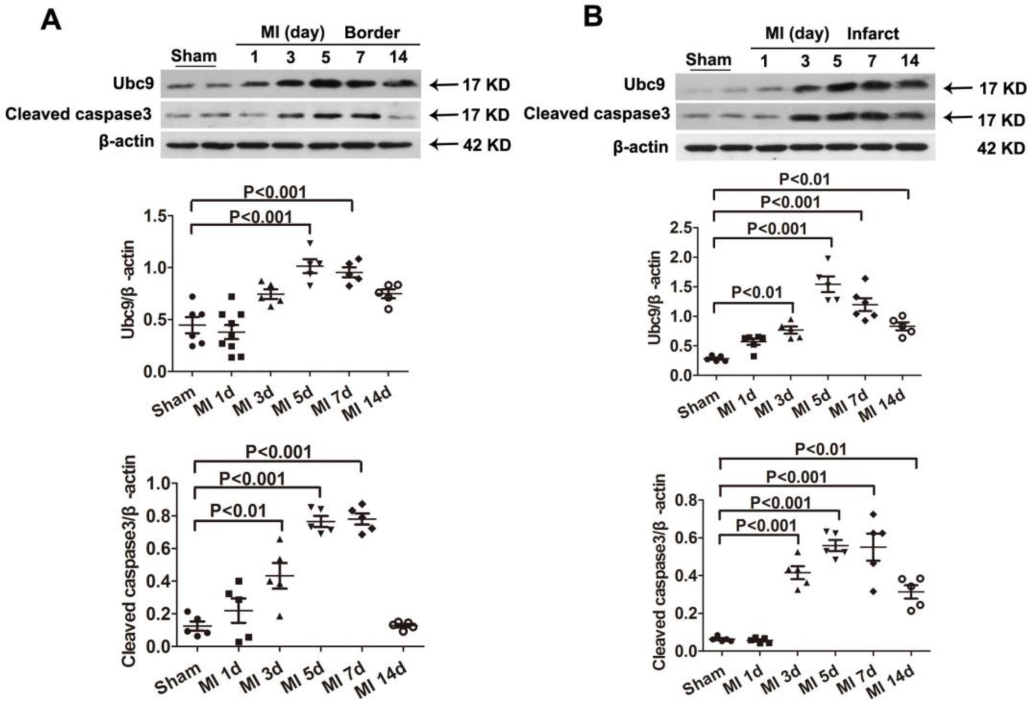

Figure S2. The change of Ubc9 and Cleaved caspase3 under MI

(A, B) Western blotting analysis of Ubc9 and Cleaved caspase3 in the border and the infarct area at different time points post-MI, n=5-9 per group. Data were analyzed by one-way ANOVA, followed by a Bonferroni post-hoc test.

Figure S3

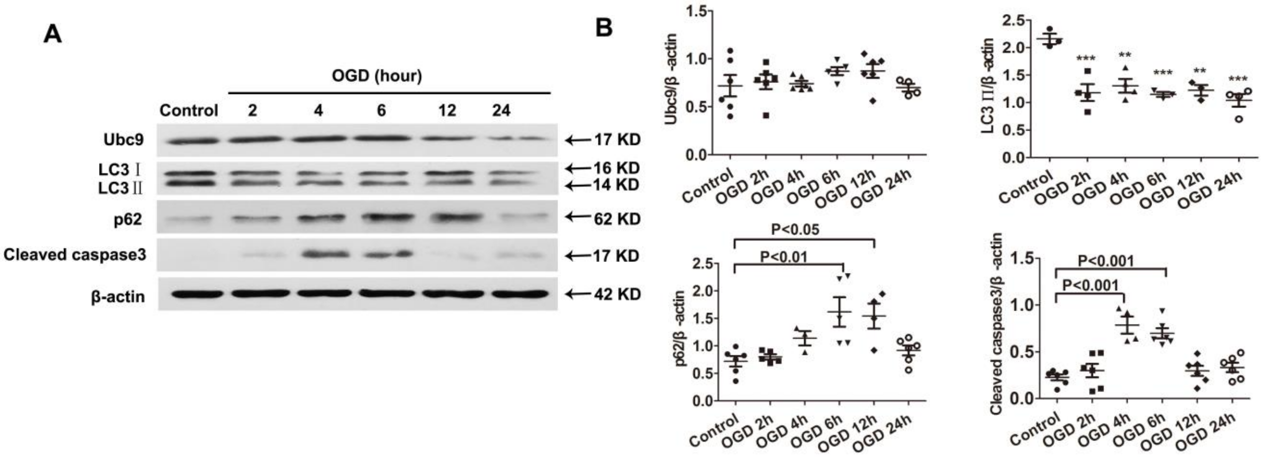

Figure S3. The change of Ubc9, Cleaved caspase3 and autophagy markers under OGD

NRCMs were treated with OGD for 24h. (A, B)Western blotting analysis of Ubc9, Cleaved caspase3 and autophagy marker LC3Ⅱ and p62 at different time points after OGD, *P<0.05, **P<0.01, ***P<0.001 versus control group, n=3-6 per group. Data were analyzed by one-way ANOVA, followed by a Bonferroni post-hoc test.

Figure S4

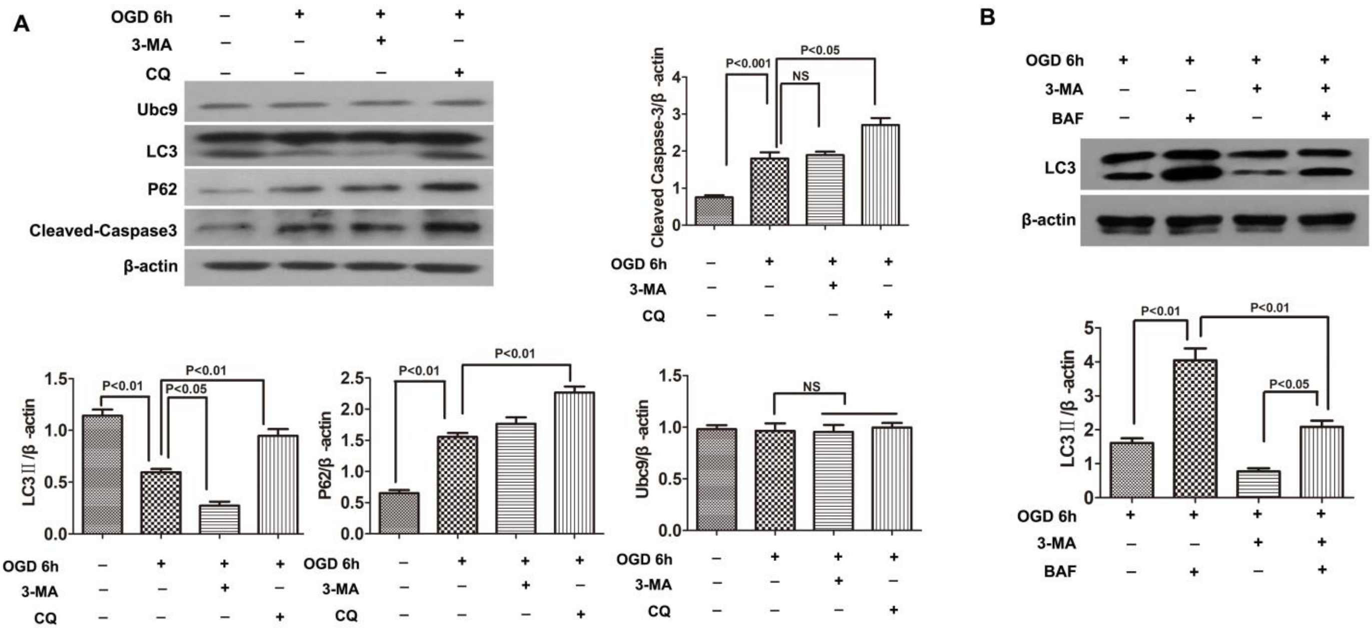

Figure S4. The effect of 3-MA and CQ on OGD-induced cardiomyocytes.

NRCMs were pre-treated with 3-MA (5 mM) or CQ (10 μM) for 12 h, or BAF (50 nM) for 2 h, and then underwent OGD for 6 h. Western blotting analysis of Ubc9, Cleaved caspase3 and autophagy marker LC3Ⅱ and p62, n=3 per group. Data were analyzed by one-way ANOVA, followed by a Bonferroni post-hoc test.

Figure S5

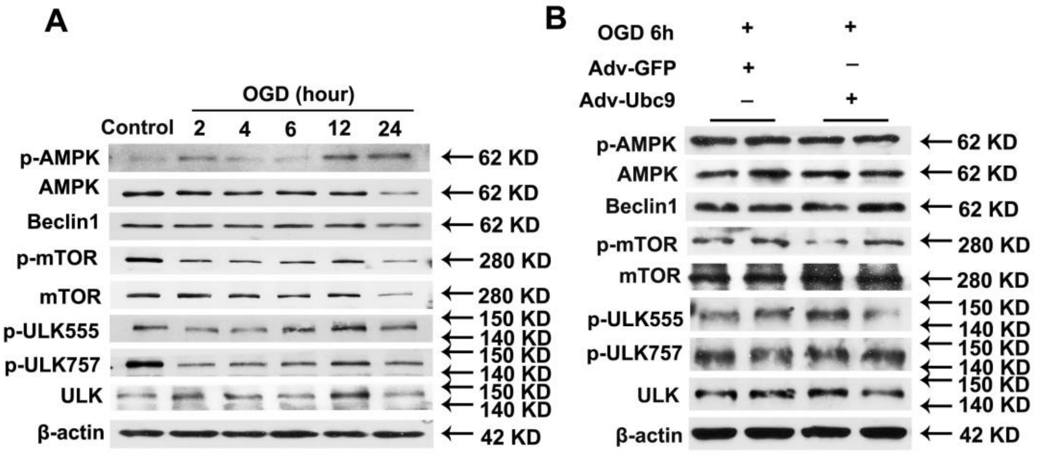

Figure S5. Ubc9 has no significant impact on the classic upstream molecules of autophagosome formation

Adv-Ubc9 was transfected in NRCMs 48 h, then maintained in hypoxic condition for 6 h. (*A*, *B*) Representative images of p-AMPK, AMPK, Beclin1, p-mTOR, mTOR, p-ULK555, p-ULK757, ULK of NRCMs by western blotting assay.

Figure S6

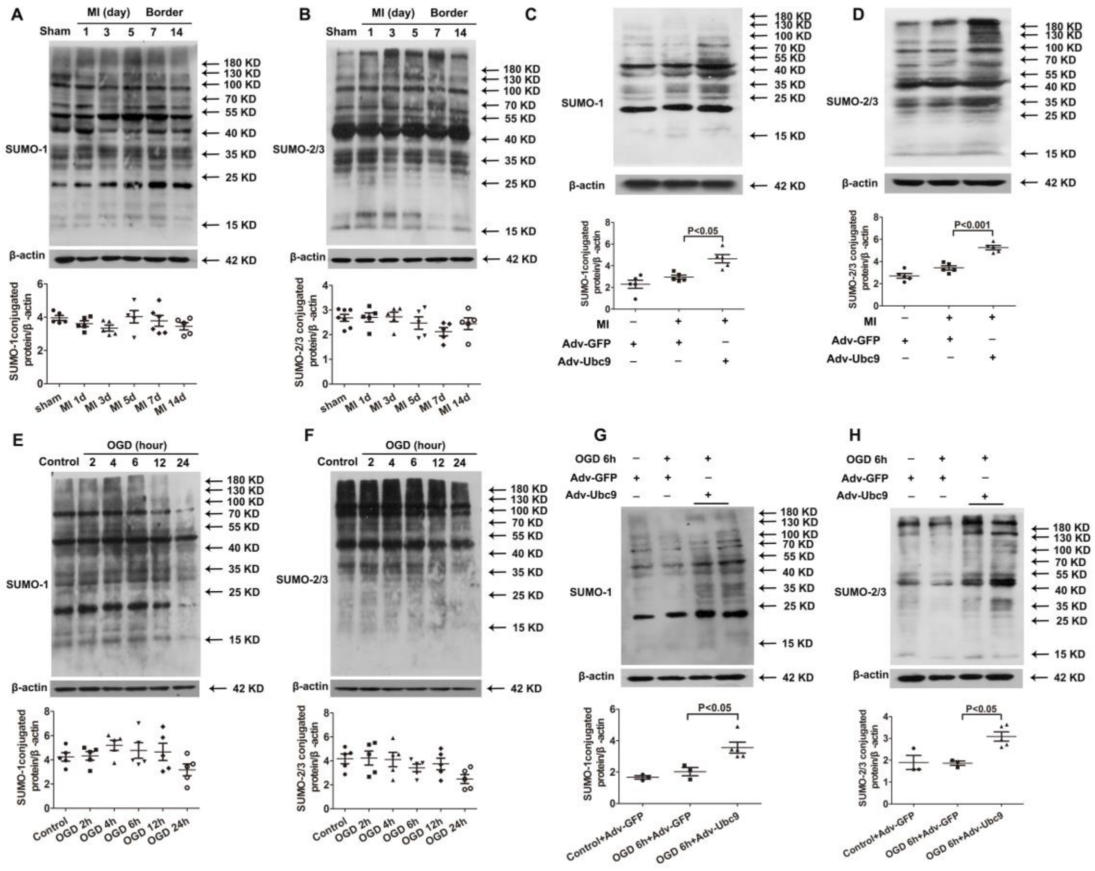

Figure S6. Ubc9 increases cardiac SUMOylated protein levels under ischemia

(*A*, *B*) Western blotting analysis of globally SUMO-1 and SUMO-2/3 conjugated protein in the border area at different time points post-MI, n=5-7 per group. (*C*, *D*) Adv-Ubc9 or Adv-GFP was multipoint injected around the ligature of heart in the C57BL/6 mice immediately after MI. Western blotting analysis of globally SUMO-1 and SUMO-2/3 conjugated protein in the border area at day 5 post-MI, n=5 per group. (*E*, *F*) Western blotting analysis of globally SUMO-1 and SUMO-2/3 conjugated protein of NRCMs at different time points post-OGD, n=5 per group. (*G*, *H*) Adv-Ubc9 was transfected in NRCMs 48 h, then maintained in hypoxic condition for 6 h. Western blotting analysis of globally SUMO-1 and SUMO-2/3 conjugated protein in NRCMs under OGD 6 h, n=3-5 per group. Data were analyzed by one-way ANOVA, followed by a Bonferroni post-hoc test.

Table S1

Table S1. Applying the SUMOsp 2.0 software to predict SUMO-modified site numbers of the autophagy related-proteins

Selecting the target proteins (*) by the characteristics of having large numbers of SUMO-modified sites or having small numbers of SUMO-modified sites while being classic in autophagic flux.

Protein	Function	Number of SUMO-modified sites
**Autophagosome formation**		
Vps34*	Part of the class Ⅲ PI3K complex, has important signal transduction in autophagosome formation and autolysosome degradation	8
Vps15*		8
Beclin1*		4
ATG9A	Transmembrane protein that delivers membrane material to the phagophore	3
Tecpr1	Full name: tectonin β-propeller repeat containing protein 1, interacts with ATG5 to activate autophagosome formation; and regulates autophagic degradation by localizing to mature autophagosomes and lysosomes	2
ATG16L1	Conjugates to ATG12-ATG5 form E3-like complex to couple LC3 to the phagophore	2
ATG16L2		2
WIPI1	Binds with PI3P recruits ATG12-ATG5-TG16L E3-like complex to the phagophore; recruits ATG9 to autophagosome membranes	0
WIPI2		2
WIPI3		0
ULK1	Serine kinase that phosphorylates autophagic components to initiate autophagy	2
ULK2		2
RAB5	The special RAB GTPase and main regulator to endocytic pathway; recruits and activates Vps34 on early endosomes and regulates early endosome membrane dynamics	2
ATG14L	Part of the class PI3K-Ⅲ complex Ⅰ and expanding phagophore; Promotes and stabilizes SNAREs of autophagosome fusion	2
ATG2A	recruits ATG9 to expanding phagophore	2
ATG2B		0
ATG10	E2-like enzyme that binds ATG12 to ATG5	1
ATG7	E1-like enzyme that binds ATG12 to ATG5 and activates LC3	1
LC3A ,B		0

	Combine with phagophore and as autophagosome marker	
Gabarapl2		0
ATG4A	Cysteine protease that activates and depletes LC3	0
ATG4B		1
ATG4C		1
ATG4D		0
ATG5	E3-like enzyme binds to ATG12 and regulates LC3 lipidation	0
ATG12	Binds to ATG5 and regulates LC3 lipidation	0
ATG13	Binds to ULK, ATG101 and FIP200 and increases activity of ULK kinase	0
ATG3	E2-like enzyme and regulates LC3 lipidation	0
ATG101	Binds to ULK, ATG101 and ATG13, regulates the ATG proteins	0
**Autophagosome-lysosome fusion**		
Vps34*	Part of the Class Ⅲ PI3K complex, has important signal transduction in autophagosome formation and autolysosome degradation	8
Vps15*		8
Vps16	A HOPS subunit promotes autophagosome and lysosome fusion	6
Beclin1*	Part of the Class Ⅲ PI3K complex, has important signal transduction in autophagosome formation and autolysosome degradation	4
RAB7	On the autophagosome, regulates autophagosome and lysosome fusion , can be induced by RAPA	3
STX17	Core SNARE family subunits, composition of STX17-Snap29-VAMP8 complex, regulates the fusion of autophagosome and lysosome	3
SNAP29	Core SNARE family subunits, composition of STX17-Snap29-VAMP8 complex, regulates the fusion of autophagosome and lysosome	3
Vps39	A HOPS subunit binds to RAB7 that recruits the HOPS complex for fusion	3
Vps41	A HOPS subunit binds to RAB7 that recruits the HOPS complex for fusion	3
UVRAG	Part of the class PI3K-Ⅲ complex Ⅱ and regulates autophagosome maturation by recruiting the core complex to autophagosome by promoting the RAB7 localization on autophagosome	3
ATG14L	Part of the Class PI3K- Ⅲ complex Ⅰ and expanding phagophore ;Promots and stablelizes SNAREs of autophagosome fusion	2
YKT6	A newly autophagosomal SNARE in fusion of autophagosome and lysosome	2
Tecpr1	Full name:tectonin β-propeller repeat containing protein 1, interacts with ATG5 to activate autophagosome formation; and regulates autophagic degradation by localizing to matural autophagosomes and	2

	lysosomes	
NAPA	Initiates ATP hydrolysis and required for SNARE function by changing structures, regulates autphagic cargo degradation	1
SNAPIN	Members of BORC recruit ARL8 and HOPS to lysosome membrane to bind with STX17 and LC3 to regulate the fusion of autophagosome and lysosome	1
KXD1		1
BLOC1S1		0
BLOC1S2		3
BORCS5		1
BORCS6		0
BORCS8		1
BORCS7		1
LC3A,B	Regulates the fusion of autophagosome and lysosome by phosphorylating PLEKHM1 and HOPS	0
Gabarapl2		0
VAMP8	Binds to STX17-SNAP29, regulates the fusion of autophagosome and lysosome	0
Vti1b	Combines with VAMP8 to promote the fusion of autophagosome and lysosome	0
PLEKHM1	Regulates autophagosome-lysosome fusion by interacting with HOPS and ATG8 family proteins	0

Supplementary Methods

Antibodies and reagents

Regent or Resource	Source	Identifier
Antibodies		
Rabbit monoclonal anti-Ubc9	Cell Signaling Technology	Cat#:4786
Rabbit polyclonal anti-p62/SQSTM1	Cell Signaling Technology	Cat#:5114
Rabbit monoclonal anti-LC3A/B	Cell Signaling Technology	Cat#:12741
Rabbit polyclonal anti-SUMO-1	Cell Signaling Technology	Cat#:4930
Rabbit monoclonal anti-SUMO-2/3	Cell Signaling Technology	Cat#:4971
Rabbit monoclonal anti-Cleaved caspase-3	Cell Signaling Technology	Cat#:9664
Rabbit monoclonal anti-Phospho-mTOR (Ser2448)	Cell Signaling Technology	Cat#:5536
Rabbit polyclonal anti-mTOR	Cell Signaling Technology	Cat#:2972
Rabbit monoclonal anti-Phospho-AMPKα (Thr172)	Cell Signaling Technology	Cat#:2535
Rabbit monoclonal anti-AMPKα	Cell Signaling Technology	Cat#:5832
Rabbit monoclonal anti-Phospho-ULK1 (Ser555)	Cell Signaling Technology	Cat#:5869
Rabbit polyclonal anti-Phospho-ULK1 (Ser757)	Cell Signaling Technology	Cat#:6888
Rabbit polyclonal anti-ULK1	Cell Signaling Technology	Cat#:4773
Mouse monoclonal anti-PI3-kinase p100 (Vps34)	Santa Cruz	Cat#:SC-365404
Rabbit polyclonal anti-Beclin1	Santa Cruz	Cat#:SC-11427
Rabbit monoclonal anti-p62/SQSTM1	Abcam	Cat#:ab109012
Rabbit polyclonal anti-β-actin	Bioworld	Cat#:AP0060
Peroxidase-Conjugated Goat anti-Mouse IgG (H+L)	ZSGB-BIO	Cat#:ZB-2305
Peroxidase-Conjugated Goat anti-Rabbit IgG (H+L)	ZSGB-BIO	Cat#:ZB-2301
Rabbit IgG	Proteintech	Cat#:B900610
normal mouse IgG	Santa Cruz	Cat#:SC-2025
Rabbit monoclonal anti-RAB7	Abcam	Cat#:ab137029
Rabbit polyclonal anti-STX17	Genetax	Cat#:GTX130212
Rabbit monoclonal anti-Snap29	Abcam	Cat#:ab138500
Rabbit monoclonal anti-Lamp1	Abcam	Cat#:ab208943
Rabbit polyclonal anti-ATG14L	Genetax	Cat#:GTX132468
Rabbit monoclonal anti-PI3 Kinase regulatory subunit 4 (Vps15)	Abcam	Cat#:ab128903
Mouse monoclonal anti-UVRAG	Santa Cruz	Cat#:SC-293268
Rabbit polyclonal anti-FYCO1	Affinity Biosciences	Cat#:AF0388
Goat anti-Rabbit IgG (H+L),Dylight 488	Abbkine	Cat#:A23220
Goat anti-Mouse IgG (H+L),Dylight 594	Abbkine	Cat#:A23410
Mouse Anti-rabbit IgG (Conformation Specific) (L27A9) mAb (HRP Conjugate)	Cell Signaling Technology	Cat#:5127
Oligonucleotides		

Ubc9-siRNA	Thermo Fisher Scientific	Cat#:4390771
NC-siRNA	Thermo Fisher Scientific	Cat#:4390843
Adenovirus		
HBAD-mRFP-GFP-LC3	Hanbio Biotechnology	
GFP-LC3	Hanbio Biotechnology	
Adv-Ubc9 NM_013050.1(rat)	Vigenebio	
Adv- Ubc9 NM_001177610 (mouse)	Vigenebio	
Chemicals， Biochemical substance		
0.25% trypsin	Solarbio	T1350
Collagenase Type II	Thermo Fisher Scientific	Cat#:17101-015
D-glucose Dulbecco′s modified Eagle medium (DMEM), High Glucose	Thermo Fisher Scientific	Cat#:10569010
Glucose and sodium pyruvate free DMEM	Sigma-Aldrich	D5030
Fetal Bovine Serum	Thermo Fisher Scientific	Cat#:10270-106
OPTI-MEM®1 Reduced Serum Medium	Thermo Fisher Scientific	Cat#:31985-062
Lipofectamine™ RNAiMAX Transfection Reagent	Thermo Fisher Scientific	Cat#:13778-150
Bafilomycin A1(BAF)	Selleck Chemicals	S1413
3-Methyladenine（3-MA）	Sigma-Aldrich	M9281
MG-132	Selleck Chemicals	S2619
Chloroquine diphosphate salt (CQ)	Sigma-Aldrich	C6628
PROTEOSTAT ® Aggresome Detection kit	ENZO Life Sciences	ENZ-51035-0025
In Situ Cell Death Detection Kit of TMR red (TUNEL)	Roche	12156792910
Annexin V-APC/7AAD Apoptosis Detection Kit	MultiSciences (LiankeBio)	AP105-100-KIT
DAPI	Beyotime	C1005
Goat serum	Bioss	C-0005
EDTA	BIOFROXX	1340GR100
Protein A/G plus-agarose	Santa Cruz	Cat#:SC-2003
RIPA Buffer (10×)	Cell Signaling Technology	Cat#:9806S
Cell Lysis Buffer (10×)	Cell Signaling Technology	Cat#:9803
Cell Lysis Buffer for Western and IP	Beyotime	P0013
Phosphatase Inhibitor Cocktail	Bimake	B15002
Protease Inhibitor Cocktail	Bimake	B14002
Tween-20	Solarbio	T8220
20× LumiGLO® Reagent and 20× Peroxide	Cell Signaling Technology	Cat#:7003
TEMED	Sigma-Aldrich	T0761
DifcoTM Skim Milk	BD	232100
1.5M Tris-HCl, PH8.8	Beyotime	ST789
1M Tris-HCl, PH6.8	Beyotime	ST768
Acr-Bis (30%, 29:1)	LEAGENE	PE0103
Pierce™ BCA Protein Assay Kit	Thermo Fisher Scientific	Cat#:23225
PBS	ORIGENE	ZLI-9063
PMSF	BEIJING DINGGUO BIOLOGY	WB-0131

Echocardiography

Transthoracic echocardiography was manipulated with a Visual Sonics (Vevo 2100; Visual Sonics Inc., Ontario, Canada) equipped with a 25-MHz imaging transducer on 14 days before sacrificing the mice. The anesthesia of the mice was kept with 2 % isoflurane gas with an inflow rate of 0.5-1.5 ml/min during the echocardiographic examination. The mice body temperature was monitored at 36-38 ℃ using a rectal thermometer, and the heart rate was maintained at 350-450 beats/min. The left ventricle (LV) was analyzed under parasternal long- axis and short-axis views, and then the output values of mice heart such as EF (ejection fraction), FS (fractional shortening), LVIDd (LV internal diameter at end-diastole) and LVIDs (LV internal diameter at end-systolic) were calculated according to the guidelines of the Vevo 2100.

TUNEL staining

At 5 days post-MI, adult male C57/B6 mice were euthanized by an overdose of sodium pentobarbital (100 mg/kg). Cell survival in ischemic area was determined by TUNEL staining on frozen sections (5 μm) or in vitro neonatal rat cardiomyocytes (NRCMs) as per manufacturer's instructions (Cell death detection assay, Roche, Indianapolis, IN). Cardiomyocytes were stained by α-actinin, and the total number of nuclei were stained by DAPI. In vivo, apoptotic cells were collected under fluorescence microscope (Nikon T1); In vitro, five fields were captured

randomly per sample under confocal microscope (Nikon American Inc., Melville, NY).

Masson's trichrome staining

On day 14 after MI, adult male C57/B6 mice were euthanized by an overdose of sodium pentobarbital (100 mg/kg). The fibrosis of myocardial tissue was determined by Masson's trichrome staining on frozen sections (5 μm). The percentage of fibrosis was analyzed by Image Pro-Plus software (Media Cybernetics Inc., Bethesda, MD, USA).

Cell culture and treatment

NRCMs were isolated as described previously.[19] Briefly, hearts from 1 to 2-day-old Sprague-Dawley rats were dissected and digested with 0.05% trypsin overnight at 4°C, then digested with Collagenase Type II for 45 minutes. cells were plated in 30mm Petri dishes and cultured in DMEM supplemented with 15% fetal bovine serum and maintained at 37°C with 5% CO_2 in a humidified chamber for 24 h, then cells changed into DMEM supplemented with 10% fetal bovine serum and cultured for 6-8 h. Subsequently, adenovirus or siRNA were delivered for 6-12 h, and then the media was replaced by fresh DMEM including 10% fetal bovine serum and continued to culture for 36-42 h. Cardiomyocytes were treated with 50 nM of bafilomycin A1 (BAF) for 2 h or 20 μM of Chloroquine (CQ) for 12 h which inhibits the fusion of autophagosomes and lysosomes, or with 5 mM 3-Methyladenine (3-MA) for 12 h which blocks

the autophagosome formation, or with 1 μM MG-132 for 24 h which restrains the ubiquitin-proteasome pathway activity before oxygen-glucose deprivation treatment (OGD), and then cells were washed twice with PBS and cultured with serum and glucose-free and sodium pyruvate-free DMEM at 37 °C in an anoxia chamber (InVivo 500, Ruskinn Life Science) saturated with 94% N_2/5% CO_2/1% O_2 for 6 h. Finally, the cardiomyocytes were harvested and analyzed.

Transfer with siRNA

NRCMs were cultured for 30 h, and then infected with 60 nM Ubc9-siRNA (Ubc9-siRNA-1, sense: CAUCCAAACGUGUAUCCUUtt; antisense: AAGGAUACACGUUUGGAUGaa. Ubc9-siRNA-2, sense: GUAUUUCAACUGCUGUAAAtt; antisense: UUUACAGCAGUUGAAAUACtg) or Negative control siRNA (NC-siRNA) using Lipofectamine™ RNAiMAX Transfection Reagent in OPTI-MEM®1 Reduced Serum Medium. After 6 h, the culture medium was changed into DMEM supplemented with 10% fetal bovine serum, and then cultured for another 42 h. In order to determine the transfected efficiency, Ubc9 expression was detected by western blot (Figure S1E), and then Ubc9-siRNA-1 was selected as the more effective one.

Ubc9 gene transfer in cardiomyocytes

In order to overexpress Ubc9, adenovirus harboring Ubc9 were infected in NRCMs as follows:

Adv-Ubc9 carrying GFP: Cells were infected with Adv-GFP at 25 multiplicity of infection (moi), or Adv-Ubc9 carrying GFP were transfected with 10, 20, 50 and 100 moi for 12 h individually. The culture medium was changed into fresh one. The cells were cultured for another 42 h before OGD treatment. Transfer efficiency of adenovirus were detected by confocal microscopy and Western blotting (Figure S1B and 1C). Adv-Ubc9 was successfully transfected at 20, 50 and 100 moi, and the efficiency was almost the same at 50 and 100 moi, so we chose 50 moi to do the subsequent experiments.

Adv-Ubc9 not carrying GFP: In order to avoid the interreference of the green fluorescence, Adv-Ubc9 not carrying GFP was used. Adv-Null (1.53×10^{13} vp/ml) or Adv-Ubc9 (1.53×10^{13} vp/ml) was delivered to cardiomyocytes as three volumes respectively: 0.05ul, 0.2ul, 0.5ul per 1ml cultural medium. The details of infection and OGD treatment were same as that used in Adv-Ubc9 carrying GFP. The transfer efficiency was confirmed by protein levels of Ubc9 and Cleaved-caspase3. Western blotting assay showed that Ubc9 was over-expressed successfully at these three volumes respectively, but Cleaved-caspase3 was down-regulated after transfected Adv-Ubc9 at the volume of 0.05 ul. In addition, cells appeared in the same status and had none obvious damage after transfecting Adv-Null or Adv-Ubc9 at the volume of 0.05 ul. Therefore, we used the concentration of 0.05 ul/ml in the subsequent experiments

(Figure S1D).

Western blot analysis

Protein preparation: Cardiomyocytes were washed three times with PBS and lysed in RIPA buffer including protease inhibitor and phosphatase inhibitor. The lysates were collected by centrifugation at 12000×g for 30 minutes at 4 °C. Then we used BCA Protein Assay Kit to determine the protein concentration, Protein samples were disposed by 5×loading buffer, and the samples were heated at 100°C for 10 minutes, then cool down in room temperature for 5 min and stored at -20˚C until required. Mouse heart was washed three times with 10 ml PBS. Isolated mice hearts were quick-frozen by liquid nitrogen and then homogenized with RIPA buffer (25 ul per mg) as described above.

Western blotting (WB): Proteins were separated in SDS-polyacrylamide gel electrophoresis (SDS-PAGE) and transferred to PVDF membrane by electrophoresis. Membranes were blocked in blocking buffer including PBS (pH 7.5), 0.1 % Tween-20 and 5 % skim milk powder for 1 h at room temperature, then membranes followed by overnight incubation with primary antibody at 4 °C as the manufactures nominated. Subsequently, membranes were washed three times (5 min per time) with 0.1% Tween 20 in PBS (PBST), then they underwent incubation with a secondary antibody conjugated with goat anti-rabbit or goat anti-mouse peroxidase-conjugated for 1 h at room temperature. After

membranes were washed four times (10 min per time) with PBST, blots were developed using 20×LumiGLO® Reagent and 20×Peroxide, molecular band intensity was determined by ImageJ.

Immunoprecipitation and immunoblotting

NRCMs were washed with PBS for three times after various treatments, and then harvested with cell lysis buffer or special SUMOylation lysis buffer (150 mM NaCl, 1 mM EDTA, 50 mM Tris-HCl, pH 7.6, 1% Triton-X-100, 2 mM PMSF, 20 mM N-ethylmaleimide (NEM), 1:100-diluted protease inhibitor cocktail). The cell lysates were cleared by centrifugation at 12000×g for 15 minutes at 4°C. Then the supernatants were incubating with 25ul protein A/G plus-agarose for 12 h at 4°C, collected supernatants by centrifugation at 2500 rpm for 15 minutes at 4°C. Added special first antibody (e.g.,anti-SUMO-1, anti-Vps34) to supernatants for immunoprecipitation (IP) 12 h at 4℃, then 25 ul protein A/G plus-agarose was added to the above mixture and incubated for 6 h at 4°C, the immune complexes were collected by centrifugation at 2500 rpm for 15 minutes at 4°C followed by washing 5 times with cell lysis buffer for Western and IP or SUMOylation lysis buffer. The immune complexes were detected by SDS-PAGE, then subjected to immunoblotting (IB) with second antibody (e.g.,anti-Beclin1, anti-RAB7), the binding state were detected by using 20×LumiGLO® Reagent and 20×Peroxide , molecular band intensity was determined by

ImageJ.

Flow cytometry

Flow cytometry was applied to detect Annexin V-APC positive apoptotic cell. The apoptotic cell was detected by the Annexin V-APC/7AAD Apoptosis Detection Kit, according to the instruction of manufacture. Briefly, the cells were stained by Annexin V-APC and 7AAD. Both early and late apoptotic cells were sorted by BD AccuriC6. Cell apoptosis was reflected by the percentage of Annexin V-APC positive cell.

Transfection of adenovirus HBAD-mRFP-GFP-LC3 /GFP-LC3

Cells were plated in confocal plate and infected with adenovirus HBAD-mRFP-GFP-LC3 with 250 moi. After 48 h, cell were treated with OGD. Confocal sections were conducted with confocal microscope (Nikon America Inc., Melville, NY) under uniform settings. The number of GFP dots (green) and mRFP dots (red) and GFP dots add mRFP dots (yellow) were calculated by fluorescent puncta from different myocytes. At least 50 cells were scored in each experiment. The number of dots per cell were received through dividing the total number of dots by the number of cells in each microscopic field. The experiments were performed in triplicate.

p62 and aggresomes colocalization assay

Cells were plated and cultured on glass coverslips. After treatments,

the slides were washed in PBS, and fixed with 4% paraformaldehyde for 20 min at room temperature. After washing with PBS three times, myocytes were permeabilized for 25 minutes with 0.1% Triton X-100 in PBS. Then cells were blocked in 10% goat serum for 1h at room temperature, and incubated overnight with p62/SQSTM1 (1:100 dilution) at 4 ℃ . The slides were incubated with second antibody of Alexa-Fluor-488-conjugated anti-rabbit IgG (1:500 dilution) for 45 minutes at room temperature. Finally, the slides were stained with the mixture of aggresomes reagent and Hoechst according to the instruction of PROTEOSTAT ® Aggresomes Detection kit. Fluorescence signals were detected using Nikon A1 laser scanning confocal microscope under uniform settings. Images were obtained and processed using the ImageJ software program. p62-Aggresomes colocalization dots were quantified by counting 50 cells per sample, and the experiments were performed in triplicate.
